# Pioglitazone and the secondary prevention of cardiovascular disease. A meta-analysis of randomized-controlled trials

**DOI:** 10.1186/s12933-017-0617-4

**Published:** 2017-10-16

**Authors:** Marit de Jong, H. Bart van der Worp, Yolanda van der Graaf, Frank L. J. Visseren, Jan Westerink

**Affiliations:** 1 Department of Vascular Medicine, University Medical Center Utrecht, PO Box 85500, 3508 Utrecht, GA The Netherlands; 2 Department of Neurology and Neurosurgery, Brain Center Rudolf Magnus, University Medical Center Utrecht, Utrecht, The Netherlands; 3 Julius Center for Health Sciences and Primary Care, University Medical Center Utrecht, Utrecht, The Netherlands; 4 Department of Vascular Medicine, University Medical Center Utrecht, Utrecht, The Netherlands

**Keywords:** Pioglitazone, Cardiovascular disease, Secondary prevention

## Abstract

**Background and aims:**

Pioglitazone targets multiple pathogenic pathways involved in the development of cardiovascular diseases (CVD). The aim of this systematic review and meta-analysis is to assess the effects of pioglitazone treatment on the secondary prevention of CVD.

**Methods:**

Randomized-controlled trials of pioglitazone in patients with CVD were identified through PubMed, Embase, Cochrane and CINAHL, in a search up to May 2016. Studies were included if pioglitazone was compared with any control (usual care, placebo or active comparator) and if patients were previously diagnosed with CVD. The outcomes of interest included major adverse cardiovascular events (MACE), myocardial infarction (MI), stroke, all-cause mortality and heart failure (HF). All outcomes were compared by pooled risk ratios (RR) with a 95% confidence interval (CI). Pooled estimates were calculated using a random-effects model.

**Results:**

Ten studies reported the effects of pioglitazone on any of the outcomes of interest. Pioglitazone reduced recurrent MACE (RR 0.74, 95% 0.60–0.92; I^2^ = 35), MI (RR 0.77, 95% CI 0.64–0.93; I^2^ = 0%), or stroke (RR 0.81, 95% CI 0.68–0.96; I^2^ = 0%). Pioglitazone did not reduce all-cause mortality (RR 0.94, 95% CI 0.81–1.08; I^2^ = 0%), whereas pioglitazone treatment was associated with an increased risk of HF (RR 1.33, 95% CI 1.14–1.54).

**Conclusions:**

Pioglitazone lowers the risk of recurrent MACE, stroke, or MI in patients with clinical manifest vascular disease. Pioglitazone does not lower the risk for all-cause mortality, and increases the risk for the development of HF.

**Electronic supplementary material:**

The online version of this article (doi:10.1186/s12933-017-0617-4) contains supplementary material, which is available to authorized users.

## Background

Patients with clinically manifest cardiovascular diseases (CVD) are at increased risk of recurrent cardiovascular events, with 28% of all stroke and coronary events combined being recurrent events [1–3]. Although a significant decrease in cardiovascular mortality has been achieved over the past decades, CVD remains the number one cause of death worldwide [3, 4]. The most important etiology for development of CVD is atherosclerosis [3]. Known modifiable risk factors for atherosclerosis include hypertension, hyperlipidemia, abdominal obesity, smoking and diabetes [5]. Insulin resistance plays an important role in the development of hypertension, hyperlipidemia and diabetes and is the hallmark feature of the metabolic syndrome which in itself is associated with an increased risk of vascular events and mortality [6, 7].

Multiple studies have suggested that pioglitazone, a peroxisome proliferator-activated receptor γ (PPARγ) agonist, used as an insulin-sensitizing agent in the treatment of type 2 diabetes mellitus (T2DM), may have anti-atherosclerotic effects [2, 8–10]. PPARγ receptors, which are mainly expressed by adipocytes and macrophages, are involved in fat adipose tissue metabolism, glucose metabolism and inflammatory processes [11–14]. Both insulin resistance and systemic low-grade inflammation are associated with atherosclerotic plaque formation and pioglitazone improves insulin resistance and reduces systemic inflammation [14–19]. In the ACT NOW trial, which studied the effects of pioglitazone on the risk of diabetes and on cardiovascular risk factors in subjects with abnormal glucose tolerance, it was shown that the use of pioglitazone was associated with a decrease in carotid intima-media thickness (CIMT) progression, which appeared not to be solely caused by improvement of traditional risk factors [20, 21]. In support of this notion, pioglitazone is associated with improvement of endothelial function in T2DM patients independent of indirect metabolic changes, further supporting the vascular disease-modifying effects of pioglitazone [22]. The potential beneficial effects of pioglitazone on cardiovascular morbidity and mortality have however come under scrutiny after the PPARγ agonist rosiglitazone was shown to be associated with an increased risk of myocardial infarction and cardiovascular death [23]. Since then multiple studies have been conducted investigating the effects of pioglitazone in patients with manifest vascular disease [2, 10].

To qualify and quantify the available data on the effect of pioglitazone on recurrent cardiovascular events in patients with manifest CVD, we performed a meta-analysis of randomized-controlled trials (RCTs) studying the effects of pioglitazone on major adverse cardiac/cardiovascular events (MACE), stroke, all-cause mortality and myocardial infarction (MI) in patients with clinical manifest CVD.

## Methods

This meta-analysis was performed according to the guidelines of the Cochrane Library using the Cochrane protocol template [24]. In addition, the quality of reporting of meta-analyses (QUOROM) was used [25].

### Search strategy and study selection

RCTs of pioglitazone in patients with CVD were identified through a search of PubMed, Embase, the Cochrane library and CINAHL (up to 10 May 2016). The complete search was re-run on September 25th (2017) to ensure that no relevant articles were missed prior to publication. Studies were included if pioglitazone was compared with any control (usual care, placebo, active comparator) for secondary prevention of cardiovascular events in patients with symptomatic CVD. All variations in treatment duration and dosage were included. The outcomes of interest included MACE, MI, stroke and all-cause mortality. Unpublished and ongoing studies were assessed by consulting http://www.clinicaltrials.gov. We used any combination of the search terms pioglitazone, CVD or intermediates, and RCTs or their synonyms in the search strategy (Additional file 1: Table S1). To identify additional eligible studies, a manual reference check was performed and Web of Science was used for citation screening. Authors where contacted when full-text data was not available. All articles were screened for relevant title/abstracts. The full text of remaining articles were independently screened by two authors (MJ and JW) after title and abstract screening. Any disagreements between these two were discussed with a third reviewer (FV). The outcomes of interest were MACE, MI, stroke or all-cause mortality. Studies were considered eligible when at least one outcome of interest was reported.

### Data extraction

The process of data extraction is detailed in Additional file 1: Table S2. Two independent authors performed the data extraction (MJ and JW); any disagreements were discussed with a third reviewer (FV). Data on heart failure (HF) was extracted post hoc from the included studies, although studies were not primarily selected for reporting this outcome.

### Quality assessment

Risk of bias for the included studies was scored by two independent authors (MJ and JW) and summarized in a ‘risk of bias graph’, including selection, performance, detection, attrition, reporting, and other bias [26, 27]. Furthermore, funnel plots were used to identify publication bias any disagreements during the quality assessment were solved consulting a third reviewer (FV).

### Data synthesis and analyses

Only dichotomous outcomes were extracted and analyzed. The extracted data was expressed as pooled risk ratios (RRs) with a 95%-confidence interval (CI 95%). The statistical heterogeneity was assessed by visual inspection of the forest plots and with the I^2^ test; an I^2^ ≥ 75% indicates considerable heterogeneity [28]. A random-effects model was used, regardless of the level of heterogeneity. Moreover, sensitivity analyses were performed comparing odds ratios (ORs) vs RRs and fixed effects models vs random effects models. Subgroup analyses were performed to study the effects of pioglitazone in patients with type 2 diabetes (T2DM) and to assess whether pioglitazone treatment differed in patients previously diagnosed with stroke. For the primary MACE endpoint we used MACE as defined in the article itself (MACE 1). We also performed a subgroup analysis on the classical 3-point MACE defined as a composite of nonfatal stroke, nonfatal MI and cardiovascular death (MACE 2). All analyses were performed using RevMan 5.2.

## Results

### Selected studies

**Fig. 1:**
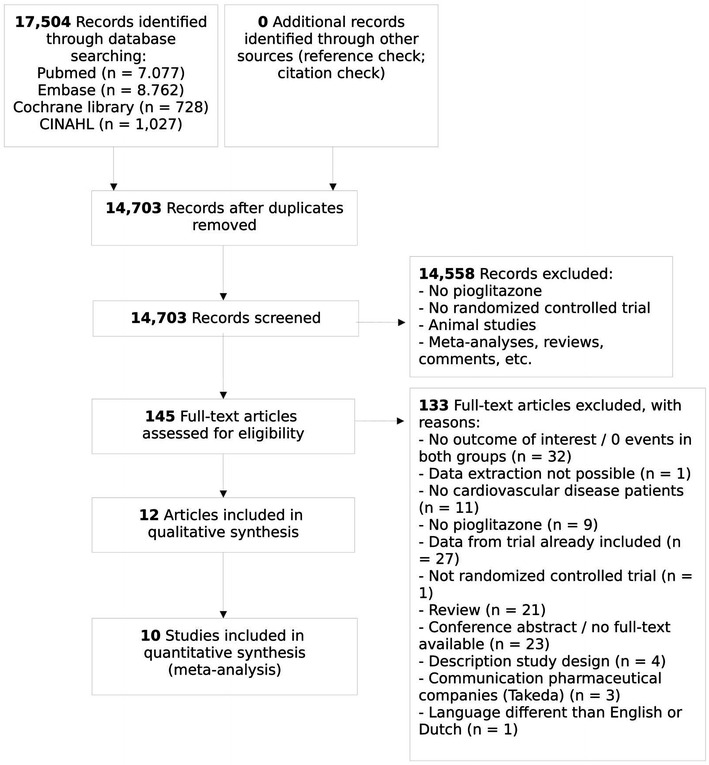
Flowchart

14,703 unique records were obtained and screened, after which 145 potentially relevant articles were selected and read in full-text. 133 Articles were excluded additionally because they did not fulfill the eligibility criteria, leaving 12 relevant articles for analysis (Fig. 1). After reference screening and citation check, no additional studies were included.

A total of ten RCTs published up to May 2016, comprising 10,252 patients were included (Table 1) [2, 8–10, 29–34]. Since data on the PROactive study was extracted from three articles, a total of twelve articles were used [10, 35, 36]. The included studies were performed over four continents. All but one study [31], reported multiple outcomes of interest and were therefore used in multiple meta-analyses. Data on MACE was extracted from eight studies [2, 9, 29, 30, 32–34, 36]. MI data was reported in eight studies [2, 9, 10, 29, 30, 32–34], stroke in four [2, 8, 9, 36], all-cause mortality in eight [2, 8, 9, 30–33, 36], and HF in seven [2, 8–10, 29, 32, 33]. Eight studies included patients with T2DM [9, 10, 29–34], of which one included patients with and without T2DM [33]. The remaining studies included patients with abnormal glucose metabolism (AGM), specifically excluding patients with known T2DM [2, 8]. In five studies, patients were included who underwent percutaneous intervention (PCI) due to significant atherosclerosis [29, 30, 32–34]. The pioglitazone dose varied from 15 mg/d up to 45 mg/day. Additional treatment in the intervention and control group consisted of standard care. The duration of treatment and follow-up varied from 2 weeks [31] up to 5 years [2, 8]. For a detailed summary of the study characteristics see Table 1 and Additional file 1: Tables S3 and S4.

**Table 1:** Study characteristics

	Total number of included studies	Hong [29]	Kaneda [33]	Kernan (IRIS) [2]	Lee [30]	Nishio [34]	Nissen (PERISCOPE) [9]	Suryadevara [31]	Takagi (POPPS) [32]	Tanaka (J-SPIRIT) [8]	Dormandy (PROactive) [10, 35, 36]
Population											
Type 2 diabetes + CVD	7	X			X	X	X	X	X		X
Type 2 diabetes + no diabetes + CVD	1		X								
Abnormal glucose metabolism without diabetes + CVD	2			X						X	
Patients undergoing PCI	5	X	X		X	X			X		
Type of pioglitazone treatment											
Pioglitazone 15 mg/d	2	X			X						
Pioglitazone 30 mg/d	3					X		X	X		
Pioglitazone 45 mg/d	1			X							
Pioglitazone 15–30 mg/d	2		X							X	
Pioglitazone 15–45 mg/d	2						X				X
Treatment in control group											
None	4		X			X			X	X	
Placebo	5	X		X	X			X			X
Glimepiride	1						X				
Extracted outcome of interest											
Major adverse c cardiac/Cardiovascular disease	8	X	X	X	X	X	X		X		X
Myocardial infarction	8	X	X	X	X	X	X		X		X
Stroke	4			X			X			X	X
All-cause mortality	8		X	X	X		X	X	X	X	X
Heart failure	6	X	X	X			X		X	X	X

*CVD* cardiovascular disease, *PCI* percutaneous coronary intervention

### Risk of bias

The risk of bias is detailed in Additional file 1: Table S5 and Figure S1. Six studies scored an unclear risk of selection bias, because of insufficient information on the random sequence generation and allocation concealment [8, 29, 30, 32–34]. Furthermore, five trials scored a high risk on performance bias, due to their study design, e.g. single-blinded or open-label [8, 29, 32–34]. Moreover, as expected all studies scoring high or unclear risk on performance bias also scored high or unclear risk on detection bias [8, 30, 32–34], with the exception of one study [29]. All but one study [31] scored low risk on attrition bias, since there was low loss to follow-up and all studies used the intention-to-treat principle [2, 8–10, 29, 30, 32–34].

### Effect of pioglitazone on major adverse cardiovascular events

**Fig. 2:**
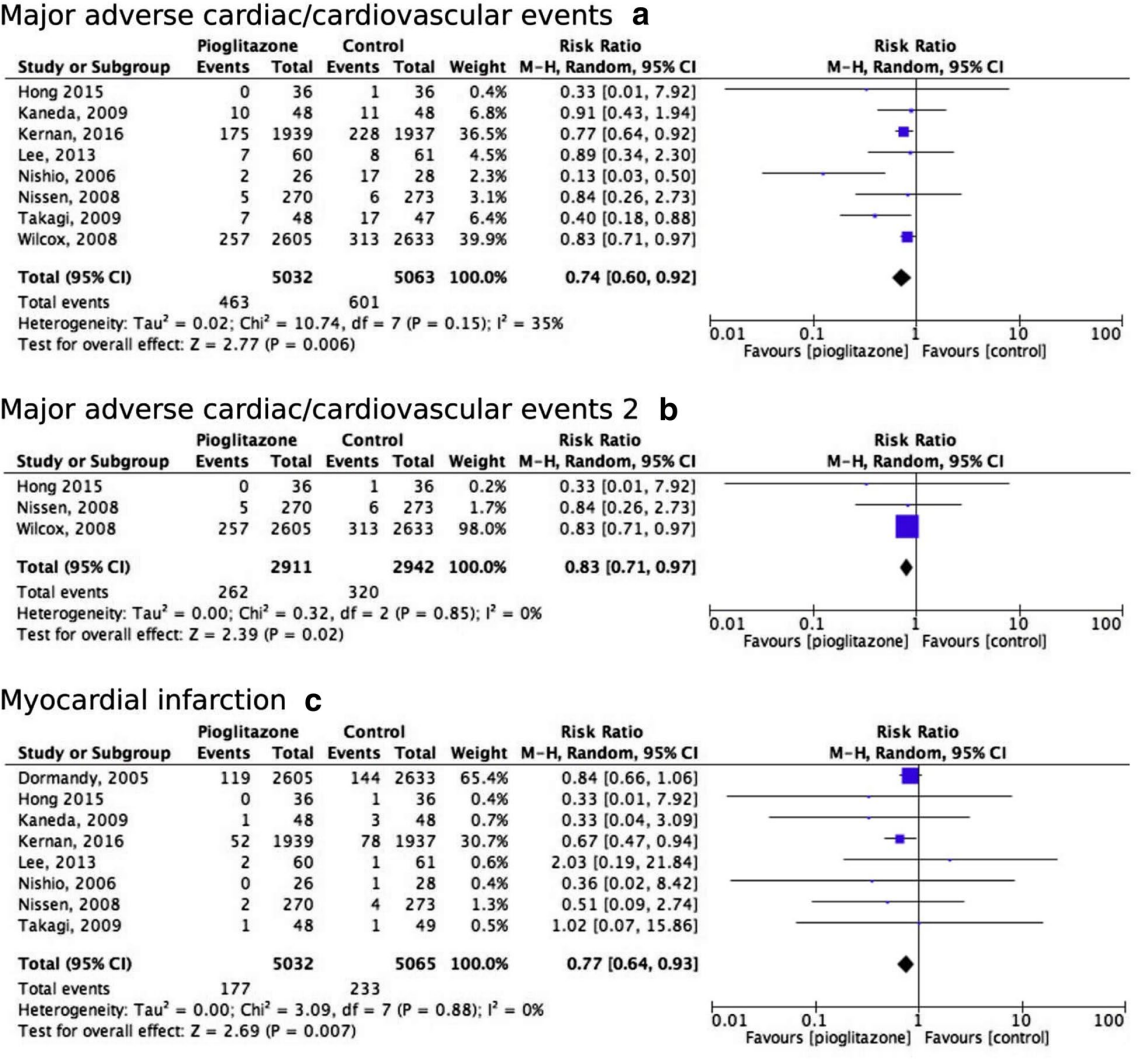
Forrest plots. Forrest plot for the effects of pioglitazone on major adverse cardiac/cardiovascular events (**a**), a composite of nonfatal myocardial infarction, nonfatal stroke and cardiovascular mortality (**b**) and myocardial infarction (**c**)

Eight studies, with a total of 10,095 participants, reported on MACE outcome and corresponding definitions and were therefore included in this analysis [2, 9, 29, 30, 32–34, 36]. Pioglitazone treatment lowered the risk of MACE compared with control with an absolute risk reduction of 2.7% (number needed to treat (NNT) 39) and a pooled RR of 0.74 (95% CI 0.60–0.92) (Fig. 2a, Additional file 1: Table S6). Moderate statistical heterogeneity was observed, I^2^ = 35% (Fig. 2a). Eyeballing of the funnel plot showed some indication of publication bias (Additional file 1: Figure S1A). Sensitivity analyses using ORs and fixed-effects models showed similar results (Additional file 1: Table S7). After exclusion of trials with patients without diabetes [33], the pooled RR was lower, with a pooled RR of 0.58 (95% CI 0.35–0.98) (Additional file 1: Table S8). In addition, a similar effect of treatment with pioglitazone was found in a subgroup analysis including studies reporting MACE as a composite of nonfatal MI, nonfatal stroke and cardiovascular mortality (MACE 2) (pooled RR 0.83, 95% CI 0.71–0.97) (Fig. 2b) [9, 29, 36. The study definitions on MACE 1 per study are summarized in Additional file 1: Table S9.

### Effect of pioglitazone on myocardial infarction

Eight studies, with a total of 10,097 participants with 410 events, reported on MI outcome and were therefore included in this analysis [2, 9, 10, 29, 30, 32–34]. It should be noted that the PERISCOPE and PROactive study only reported data on nonfatal MI [9, 10], Furthermore, the PROactive included silent MIs in their data [10]. The study definitions on MI per study are summarized in Additional file 1: Table S10. Patients receiving pioglitazone were at lower risk of MI, with an absolute risk reduction of 1.1% (NNT of 93) and a pooled RR of 0.77 (95% CI 0.64–0.93) (Fig. 2c and Additional file 1: Table S6). No statistical heterogeneity was observed, I^2^ = 0% (Fig. 2c). Furthermore, visual inspection of the funnel plot showed no indication of publication bias (Additional file 1: Figure S1B) and sensitivity analyses were comparable using ORs and fixed-effects models (Additional file 1: Table S7). Exclusion of studies with patients without diabetes [2, 33], showed little difference in the estimated RR, although the results became non-significant (pooled RR 0.83, 95% CI 0.66–1.04) (Additional file 1: Table S8).

### Effect of pioglitazone on stroke

**Fig. 3:**
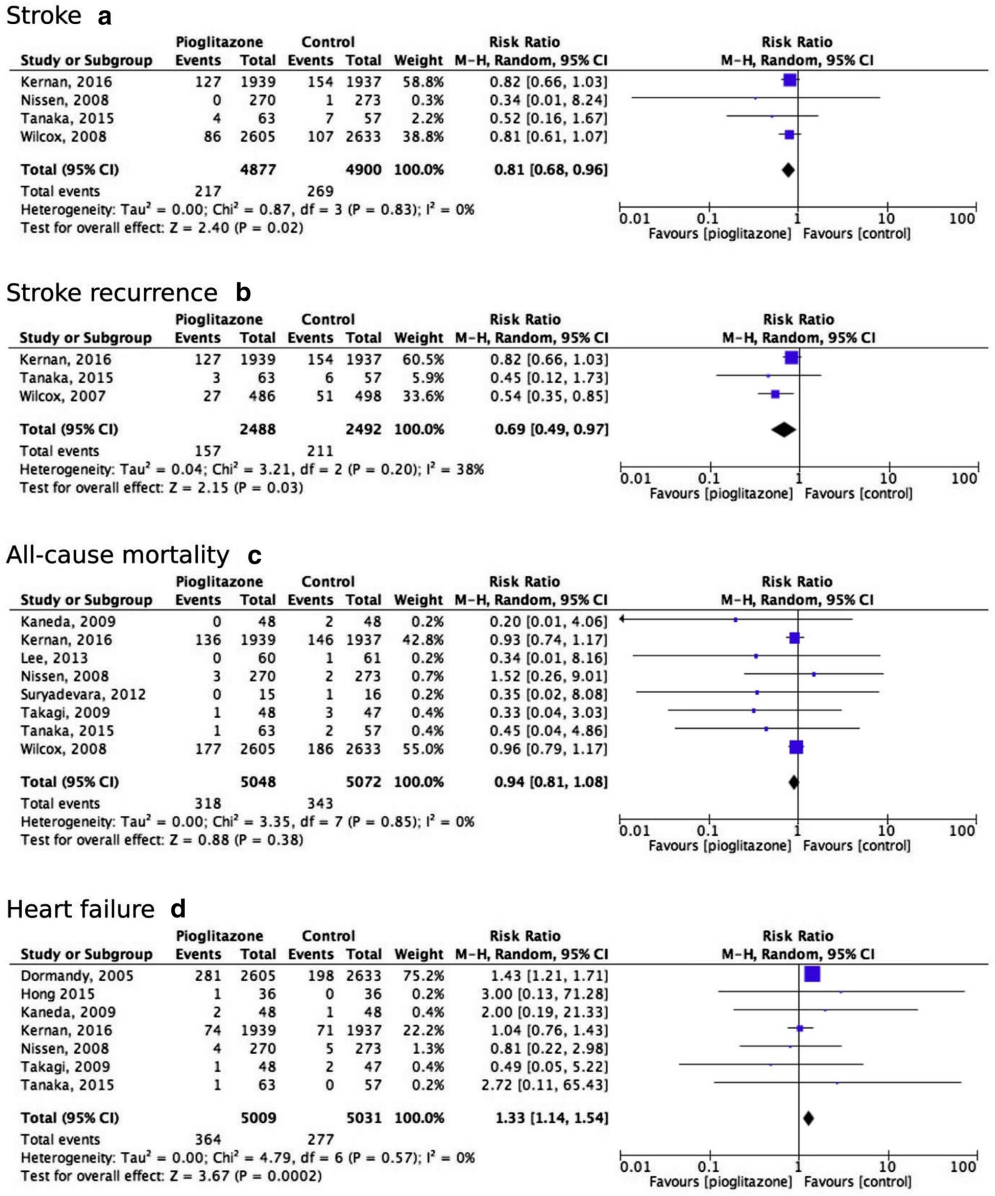
Forrest plots. Forrest plot for the effects of pioglitazone on stroke (**a**), stroke recurrence (**b**), all-cause mortality (**c**) and heart failure (**d**)

Four studies, with a total of 9777 participants with 486 events, reported on stroke and were therefore included in this analysis [2, 8, 9, 36]. The study definitions on stroke per study are summarized in Additional file 1: Table S9. The pooled RR for stroke was significantly in favor of pioglitazone treatment, with an absolute risk reduction of 1.0% (NNT 91) and a pooled RR of 0.81 (95% CI 0.68–0.96) (Fig. 3a; Additional file 1: Table S6) [2, 8, 9, 36]. However, it should be noted that the PERISCOPE study only reported nonfatal stroke [9]. No statistical heterogeneity was observed (I^2^ = 0%) and visual inspection of the funnel plot showed some indication for publication bias (Fig. 3a; Additional file 1: Figure S1C). Excluding all studies reporting data on patients without diabetes [9, 8], resulted in a pooled RR of 0.81 (95% CI 0.61–1.07) (Additional file 1: Table S8). In addition, a similar effect of treatment with pioglitazone was found in a subgroup analysis that included studies reporting on stroke recurrence in patients with a prior history of stroke (pooled RR 0.69, 95% CI 0.49–0.97) (Fig. 3b) [2, 8, 35].

### Effect of pioglitazone on all-cause mortality

For the analysis of the effect of pioglitazone on all-cause mortality, eight studies were included, with a total of 10,120 participants with 661 events [2, 8, 9, 30–33, 36]. Pioglitazone treatment did not lower all-cause mortality risk (RR 0.94, 95% CI 0.81–1.08) (Fig. 3c). There was no statistical heterogeneity (I^2^ = 0%) and visual inspection of the funnel plot showed some indication for publication bias (Fig. 3c; Additional file 1: Figure S1D). When excluding all studies with patients without diabetes [2, 8, 33], comparable results were observed (Additional file 1: Table S8). We were not able to perform a meta-analysis on cardiovascular mortality since appropriate data was lacking.

### Effect of pioglitazone on heart failure

Eight studies reported HF as adverse event [2, 8–10, 29, 32–34], of which seven were included in our analysis, with a total of 10.040 participants with 641 events [2, 8–10, 29, 32, 33]. One study was excluded because of an absence of occurrence of heart failure in both groups [34]. The study definitions on HF per study are summarized in Additional file 1: Table S12. Pioglitazone treatment lead to a higher risk of HF (pooled RR 1.33, 95% CI 1.14–1.54), with a number needed to harm (NNH) of 57 (Fig. 3d; Additional file 1: Table S6). No statistical heterogeneity was observed, I^2^ = 0% (Fig. 3d). Excluding all studies with patients without diabetes resulted in a slight increase of the estimated RR (pooled RR 1.42, 95% CI 1.19–1.68) (Additional file 1: Table S8) [2, 33].

## Discussion

### Summary of main results

In this meta-analysis on the effects of pioglitazone for secondary prevention of cardiovascular disease (CVD), pioglitazone lowered the risk of recurrent major adverse cardiac/cardiovascular events (MACE) by 26%, of stroke by 19%, and of myocardial infarction (MI) by 23%. Pioglitazone had no effect on all-cause mortality and increased the risk of heart failure (HF) by 33% in patients with clinical manifest vascular disease.

### Comparison with other studies

The results of three other meta-analyses on the effects of pioglitazone on CVD in randomized–controlled trials (RCTs), are supportive to our results in their conclusion that pioglitazone has protective effects on CVD. However the effects of pioglitazone on HF were inconsistent among the studies, which may in part be explained by differences in the study populations [37–39]. In contrast to these previous meta-analyses, we studied the effects of pioglitazone on cardiovascular events in a specific population of patients with a history of CVD, thereby not restricting our study to patients with insulin resistance, pre-diabetes or diabetes mellitus type 2 (T2DM). This is important since pioglitazone might be able to reduce the residual cardiovascular risk seen in patients with prevalent CVD with and without T2DM.

The results of the present meta-analysis show a beneficial effect of pioglitazone on the risk of recurrent cardiovascular events in patients with established CVD. In current clinical practice, pioglitazone is not widely used in secondary prevention in patients with or without diabetes, since treatment with pioglitazone is restricted to patients with T2DM and specialist have become more reluctant in prescribing pioglitazone, due to the increased risk of triggering or worsening of symptoms of HF in susceptible patients, as is also shown in our meta-analysis [40–42]. The precise mechanisms by which pioglitazone may improve cardiovascular outcomes is not completely solved, although a number of studies suggests anti-atherosclerotic effects of pioglitazone as the driver for this reduction. For example, pioglitazone has been associated with a reduction of coronary inflammation [43], alterations in the coronary atherosclerotic core composition and in particular a reduction of the necrotic core [44, 45], a reduction of neointima volume after stent implantation [46], and a decrease of carotid intima-media thickness progression [20, 21]. Furthermore, pioglitazone has been linked to improvement of endothelial function in T2DM patients, independent of indirect metabolic changes, further supporting the vascular-modifying effects of pioglitazone [22].

A recent meta-analysis of multiple safety outcomes including HF, fractures, edema and weight gain in patients with T2DM, pre-diabetes or insulin resistance with and without CVD, concluded that treatment with pioglitazone was associated with an increased risk on the development of heart failure (RR 1.32, 95% CI 1.14–1.54), fractures, edema and weight gain, while there was no significant difference in all-cause mortality between pioglitazone and control group [38]. Although pioglitazone is associated with worsening of HF or HF development, it is not associated with adverse effects on cardiac function or structure itself [47]. Interestingly, various studies showed improvement of left ventricular systolic and diastolic function in patients with T2DM during pioglitazone treatment [48–50]. Although the effect of pioglitazone on left ventricular systolic and diastolic function as a possible explanation for the increased incidence of heart failure is still a matter of debate [51, 52]. Since pioglitazone treatment is associated with development of peripheral edema, with an incidence up to 7.5% if combined with other antidiabetic drugs, it is suggested that HF may be mainly due to fluid retention rather than primarily cardiac dysfunction [47, 53]. A recent cohort study in an Asian population on the effects of thiazolidinediones on cardiovascular effects in diabetic patients without pre-existing CVD, suggested that pioglitazone use is not associated with an increased risk on development of HF (HR 0.94, 95% CI 0.59–1.50) [54]. By contrast, our meta-analysis in patients with CVD at baseline showed an increased risk of 33% for developing HF during pioglitazone use. Thus, patients with CVD using pioglitazone may be at higher risk for HF development compared to patients using pioglitazone but without CVD [54]. Finally, although not investigated in this study, other concerns still surround the use of pioglitazone in relation to malignancies, especially for bladder cancer, as well as the possibly associated risk of osteoporosis and fractures [55–61]. These findings have led current guidelines to restrict the use of pioglitazone in specific subgroups of patients, most importantly in patients with symptomatic heart disease or bladder cancer [47, 61, 62]. Whether pioglitazone is still efficacious in reducing cardiovascular events and can be safely used in patient populations who are at a lower risk for developing heart failure is currently unknown as studies are lacking.

Based on the data from this meta-analysis, further studies are needed to investigate whether pioglitazone might be superior to other glucose lowering drugs, especially in patients with CVD who might benefit the most. Direct comparison in trials between pioglitazone and other glucose lowering drugs on cardiovascular morbidity and mortality is limited to the recently published TOSCA.IT, which compared pioglitazone and sulfonylureas and found no difference on cardiovascular events. Data from a nationwide cohort in Taiwan suggests that patients on pioglitazone are at a lower risk for cardiovascular morbidity than patients on DDP4 inhibition. However, these data should be interpreted with caution as considerable confounding by indication is evident [63]. In addition, use of pioglitazone in patients with symptomatic HF or susceptible patients is not recommended and the use of pioglitazone is contra-indicated in patients with established NYHA class III or IV heart failure [61]. Moreover, the association between pioglitazone and urinary bladder tumors is still a matter of debate and the use of pioglitazone in patients with active bladder is therefore not recommended [61].

### Study limitations

Several study limitations of this meta-analysis should be considered. First there was some methodological heterogeneity between the studies, including the individual objectives, study populations, risk factors for development and progression of CVD, heterogeneity on the definitions of the outcomes of interest, in particular for HF and MACE, dosages of pioglitazone, types of control, treatment, and follow-up duration. Overall, differences across the individual studies were observed for medical history and a number of cardiovascular risk factors, including BMI, HbA1c, smoking status, presence of hypertension, systolic blood pressure, LDL-cholesterol, total cholesterol and medication use, as can be seen in Additional file 1: Table S4. These differences should be taken into account when interpreting the results of this meta-analysis. For example, cardiovascular risk management between the two major trials of this meta-analysis—PROactive and IRIS—are quite different. In the IRIS trial more strict treatment of risk factors was evident with 70% of participants on statins compared to 40% in the PROactive study. Furthermore, there is difference of ~ 10 mmHg in systolic blood pressure in favor of the IRIS trial (Additional file 1: Table S4). Nevertheless, both trials indicate a lower risk for recurrent cardiovascular events by pioglitazone [2, 10]. In addition, heterogeneity was observed for duration of follow-up and treatment. Most of the studies with a follow-up duration shorter than 12 months are however small studies with a subsequent low number of events [29, 31, 33, 34]. Consequently, the weight of these studies in the meta-analyses is quite small and therefore they do not have a large effect on the overall results of the meta-analyses, as can been in Additional file 1: Figures S2–S6, after stratifying the included studies on follow-up of 12 months or longer. Moreover, the study from Suryadevara et al. from 2012 was somewhat different compared to the other included studies. Although the participants from this study differ on some aspects from the other included studies, for example 100% of the participants are hypertensive and all of them took statins, it is not likely that these differences affect the results of the meta-analyses and the interaction between pioglitazone and the outcomes of interest, since the weight of this study on the meta-analyses is quite small [31]. Also, a number of studies included participants with recent percutaneous coronary intervention (PCI) [29, 30, 32–34]. Since the studies with participants that underwent recent PCI are limited in number of participants and events, separate analyses on CVD outcomes in participants that underwent recent PCI were not performed. Although it would be interesting for future research, to study whether patients on pioglitazone with recent PCI have different CVD outcomes compared to patients on pioglitazone with coronary artery disease (CAD) without recent PCI. In some of the included studies treatment duration versus follow-up duration was not reported [8, 29, 34]. We assumed that treatment duration was equal to follow-up duration when studies only mentioned follow-up duration. Furthermore, publication bias could not entirely be excluded, since less than ten studies were included in the individual analysis. The methodological quality for the included studies was variable and the risk of bias among the included studies should be taken into account when interpreting the results of this meta-analysis.

## Conclusions

In conclusion, pioglitazone lowers the risk of recurrent MACE, stroke and MI in patients with clinical manifest vascular disease. Pioglitazone does not lower the risk of all-cause mortality during the study periods, and increases the risk for the development of HF.

## Additional file

**Additional file 1: Table S1.** Search strategy + date performed. **Table S2.** Details on data extraction. **Table S3.** Additional study characteristics. **Table S4.** Additional study characteristics – modifiable risk factors + medication. **Table S5.** Risk of bias summary. **Table S6.** Absolute risk reduction, numbers needed to treat/harm and relative risk. **Table S7.** Sensitivity analyses. **Table S8.** Subgroup analyses. **Table S9.** Definitions of major adverse cardiac/cardiovascular events. **Table S10.** Definitions of myocardial infarction. **Table S11.** Definitions of Stroke. **Table S12.** Definitions of heart failure. **Figure S1.** Funnel plots for major adverse cardiac/cardiovascular events (**a**), myocardial infarction (**b**), stroke (**c**), and all-cause mortality (**d**). *MACE* major adverse cardiac/cardiovascular events, *MI* myocardial infarction. **Figure S2.** Analysis on major adverse cardiac/cardiovascular events. Only studies with a follow-up duration of 12 months or longer were included. **Figure S3.** Analysis on major adverse cardiac/cardiovascular events 2. Only studies with a follow-up duration of 12 months or longer were included. **Figure S4.** Analysis on myocardial infarction. Only studies with a follow-up duration of 12 months or longer were included. **Figure S5.** Analysis on all-cause mortality. Only studies with a follow-up duration of 12 months or longer were included. **Figure S6.** Analysis on heart failure. Only studies with a follow-up duration of 12 months or longer were included.

## Abbreviations

AGM: abnormal glucose metabolism
CAD: coronary artery disease
CI: confidence interval
CIMT: carotid intima-media thickness
CVD: cardiovascular diseases
HF: heart failure
MACE: major adverse cardiovascular events
MACE 1: major adverse cardiovascular events as defined in the article itself
MACE 2: major adverse cardiovascular events as a composite if nonfatal stroke nonfatal MI and cardiovascular death
MI: myocardial infarction
NNH: number needed to harm
NNT: number needed to treat
ORs: odds ratios
PCI: percutaneous intervention
PPARγ: peroxisome proliferator-activated receptor γ
QUOROM: quality of reporting of meta-analyses
RCTs: randomized-controlled trials
RRs: risk ratios
T2DM: type 2 diabetes mellitus
TIA: transient ischemic attack
