# Supplementary material for: Pioglitazone and the secondary prevention of cardiovascular disease. A meta-analysis of randomized-controlled trials
Source: Cardiovasc Diabetol. 2017 Oct 16;16:134. doi: 10.1186/s12933-017-0617-4 (PMC5644073; doi:10.1186/s12933-017-0617-4)
Supplement: Supplementary file 1 — Additional file 1: Table S1. Search strategy + date performed. Table S2. Details on data extraction. Table S3. Additional study characteristics. Table S4. Additional study characteristics – modifiable risk factors + medication. Table S5. Risk of bias summary. Table S6. Absolute risk reduction, numbers needed to treat/harm and relative risk. Table S7. Sensitivity analyses. Table S8. Subgroup analyses. Table S9. Definitions of major adverse cardiac/cardiovascular events. Table S10. Definitions of myocardial infarction. Table S11. Definitions of Stroke. Table S12. Definitions of heart failure. Figure S1. Funnel plots for major adverse cardiac/cardiovascular events (a), myocardial infarction (b), stroke (c), and all-cause mortality (d). MACE major adverse cardiac/cardiovascular events, MI myocardial infarction. Figure S2. Analysis on major adverse cardiac/cardiovascular events. Only studies with a follow-up duration of 12 months or longer were included. Figure S3. Analysis on major adverse cardiac/cardiovascular events 2. Only studies with a follow-up duration of 12 months or longer were included. Figure S4. Analysis on myocardial infarction. Only studies with a follow-up duration of 12 months or longer were included. Figure S5. Analysis on all-cause mortality. Only studies with a follow-up duration of 12 months or longer were included. Figure S6. Analysis on heart failure. Only studies with a follow-up duration of 12 months or longer were included. [file 12933_2017_617_MOESM1_ESM.docx]

**Additional material**

**Figure Legends**

**Figure S1.** Funnel plots

Funnel plots for major adverse cardiac/cardiovascular events (A), myocardial infarction (B), stroke (C), and all-cause mortality (D). MACE=major adverse cardiac/cardiovascular events; MI=myocardial infarction.

**Figure S2.** Analysis on major adverse cardiac/cardiovascular events

Only studies with a follow-up duration of 12 months or longer were included.

**Figure S3.** Analysis on major adverse cardiac/cardiovascular events 2

Only studies with a follow-up duration of 12 months or longer were included.

**Figure S4.** Analysis on myocardial infarction

Only studies with a follow-up duration of 12 months or longer were included.

**Figure S5.** Analysis on all-cause mortality

Only studies with a follow-up duration of 12 months or longer were included.

**Figure S6.** Analysis on heart failure

Only studies with a follow-up duration of 12 months or longer were included.

**Additional Table S1:** Search strategy + date performed

| Pubmed – 10 May |
| --- |
| Search (((((Pioglitazone[Title/Abstract] OR thiazolidinedione[Title/Abstract] OR thiazolidinediones [Title/Abstract] OR glitazone[Title/Abstract] OR glitazones[Title/Abstract] OR 2,4-thiazolidinedione[Title/Abstract] OR 2,4-thiazolidinediones[Title/Abstract] OR Actos[Title/Abstract] OR AD4833[Title/Abstract] OR U72107A[Title/Abstract] OR “Peroxisome proliferator-activated receptor gamma agonist”[Title/Abstract] OR “Peroxisome proliferator-activated receptor gamma”[Title/Abstract] OR “PPAR gamma”[Title/Abstract] OR PPARgamma[Title/Abstract] OR PPAR-gamma[Title/Abstract] OR PPARG[Title/Abstract] OR NR1C3[Title/Abstract] OR PPAR[Title/Abstract]))) AND ((randomized controlled trial[pt] OR controlled clinical trial [pt] OR randomized [tiab] OR placebo [tiab] OR clinical trials as topic [mesh: noexp] OR randomly [tiab] OR trial [ti])))) OR ((((((((((((((("Abdominal aortic aneurysm”[Title/Abstract] OR “abdominal aorta aneurysm”[Title/Abstract]))) OR ((Embolism[Title/Abstract] OR thrombosis[Title/Abstract]))) OR (("ankle brachial index"[Title/Abstract] OR "ankle brachial ratio"[Title/Abstract] OR "arterial stiffness"[Title/Abstract] OR "vascular stiffness"[Title/Abstract] OR "pulse wave analysis"[Title/Abstract] OR "intima media thickness"[Title/Abstract] OR "pulse velocity"[Title/Abstract] OR "pulse wave velocity"[Title/Abstract] OR "augmentation index"[Title/Abstract] OR "aortic calcification"[Title/Abstract]))) OR ((death[Title/Abstract] OR mortality[Title/Abstract] OR survival[Title/Abstract]))) OR ((“percutaneous coronary intervention"[Title/Abstract] OR "percutaneous transluminal angioplasty"[Title/Abstract] OR "percutaneous transluminal coronary angioplasty"[Title/Abstract] OR "coronary artery bypass graft"[Title/Abstract] OR "carotid endarterectomy"[Title/Abstract] OR "carotid artery stenting"[Title/Abstract]))) OR ((“peripheral artery disease"[Title/Abstract] OR "peripheral arterial disease"[Title/Abstract] OR "peripheral artery occlusive disease"[Title/Abstract] OR PAD[Title/Abstract] OR atherosclerosis[Title/Abstract] OR "arterial calcification"[Title/Abstract] OR atherogenesis[Title/Abstract]))) OR ((stroke[Title/Abstract] OR poststroke[Title/Abstract] OR post-stroke[Title/Abstract] OR "cerebrovascular disease"[Title/Abstract] OR "cerebrovascular event"[Title/Abstract] OR "cerebrovascular ischemia"[Title/Abstract] OR "cerebrovascular ischaemia"[Title/Abstract] OR "cerebrovascular accident"[Title/Abstract] OR "cerebrovascular hemorrhage" [Title/Abstract] OR “cerebrovascular disorders”[Title/Abstract] OR “cerebrovascular trauma” [Title/Abstract] OR “cerebral hematoma”[Title/Abstract] OR “brain hematoma”[Title/Abstract] OR hematoma[Title/Abstract] OR "cerebrovascular infarction”[Title/Abstract] OR “intracranial arterial diseases”[Title/Abstract] OR "cerebral disease"[Title/Abstract] OR "cerebral event"[Title/Abstract] OR "cerebral ischemia"[Title/Abstract] OR "cerebral ischaemia"[Title/Abstract] OR "cerebral accident"[Title/Abstract] OR "cerebral hemorrhage"[Title/Abstract] OR "cerebral haemorrhage"[Title/Abstract] OR “intracranial hemorrhage”[Title/Abstract] OR “intracranial haemorrhage”[Title/Abstract] OR "cerebral infarction"[Title/Abstract] OR "brain infarction"[Title/Abstract] OR "brain hemorrhage"[Title/Abstract] OR "brain haemorrhage"[Title/Abstract] OR "brain ischemia"[Title/Abstract] OR "brain ischaemia"[Title/Abstract] OR “Intracranial vasospasm”[Title/Abstract] OR vasospasm[Title/Abstract] OR "transient ischemic attack"[Title/Abstract] OR "transient ischaemic attack"[Title/Abstract] OR TIA[Title/Abstract] OR TIAs[Title/Abstract] OR “carotid artery diseases”[Title/Abstract]))) OR (("coronary disease"[Title/Abstract] OR "coronary ischemia"[Title/Abstract] OR "coronary ischaemia"[Title/Abstract] OR "coronary artery disease"[Title/Abstract] OR "coronary heart disease"[Title/Abstract] OR "myocardial infarction"[Title/Abstract] OR MI[Title/Abstract] OR "heart infarction”[Title/Abstract]))) OR ((“cardiovascular disease”[Title/Abstract] OR "cardiovascular event" [Title/Abstract] OR “cardiovascular disorder”[Title/Abstract] OR “cardiovascular outcome” [Title/Abstract] OR “vascular risk” [Title/Abstract] OR “cardiovascular risk”[Title/Abstract])))) OR ((“myocardial infarction" OR stroke[MeSH Terms])))) AND ((Pioglitazone[Title/Abstract] OR thiazolidinedione[Title/Abstract] OR thiazolidinediones [Title/Abstract] OR glitazone[Title/Abstract] OR glitazones[Title/Abstract] OR 2,4-thiazolidinedione[Title/Abstract] OR 2,4-thiazolidinediones[Title/Abstract] OR Actos[Title/Abstract] OR AD4833[Title/Abstract] OR U72107A[Title/Abstract] OR “Peroxisome proliferator-activated receptor gamma agonist”[Title/Abstract] OR “Peroxisome proliferator-activated receptor gamma”[Title/Abstract] OR “PPAR gamma”[Title/Abstract] OR PPARgamma[Title/Abstract] OR PPAR-gamma[Title/Abstract] OR PPARG[Title/Abstract] OR NR1C3[Title/Abstract] OR PPAR[Title/Abstract]))) |
| Cochrane Library – 9 May |
| (Pioglitazone or thiazolidinedione or thiazolidinediones or glitazone or glitazones or 2,4-thiazolidinedione or 2,4-thiazolidinediones or Actos or AD4833 or U72107A or "Peroxisome proliferator-activated receptor gamma agonist" or "Peroxisome proliferator-activated receptor gamma" or "PPAR gamma" or PPARgamma or PPAR-gamma or PPARG or NR1C3) and ("cardiovascular disease" or "cardiovascular event" or "cardiovascular disorder" or "cardiovascular outcome" or "vascular risk" or "cardiovascular risk" or "coronary disease" or "coronary ischemia" or "coronary ischaemia" or "coronary artery disease" or "coronary heart disease" or "myocardial infarction" or MI or "heart infarction" or stroke or poststroke or post-stroke or "cerebrovascular disease" or "cerebrovascular event" or "cerebrovascular ischemia" or "cerebrovascular ischaemia" or "cerebrovascular accident" or "cerebrovascular hemorrhage" or "cerebrovascular disorders" or "cerebrovascular trauma" or "cerebral hematoma" or "brain hematoma" or hematom or "cerebrovascular infarction" or "intracranial arterial diseases" or "cerebral disease" or "cerebral event" or "cerebral ischemia" or "cerebral ischaemia" or "cerebral accident" or "cerebral hemorrhage" or "cerebral haemorrhage" or "intracranial hemorrhage" or "intracranial haemorrhage" or "cerebral infarction" or "brain infarction" or "brain hemorrhage" or "brain haemorrhage" or "brain ischemia" or "brain ischaemia" or "Intracranial vasospasm" or vasospasm or "transient ischemic attack" or "transient ischaemic attack" or TIA or TIAs or "carotid artery diseases" or "peripheral artery disease" or "peripheral arterial disease" or "peripheral artery occlusive disease" or PAD or atherosclerosis or arterial calcification or atherogenesis or "percutaneous coronary intervention" or "percutaneous transluminal angioplasty" or "percutaneous transluminal coronary angioplasty" or "coronary artery bypass graft" or "carotid endarterectomy" or "carotid artery stenting" or death or mortality or survival or "ankle brachial index" or "ankle brachial ratio" or "arterial stiffness" or "vascular stiffness" or "pulse wave analysis" or "intima media thickness" or "pulse velocity" or "pulse wave velocity" or "augmentation index" or "aortic calcification" or Embolism or thrombosis or "Abdominal aortic aneurysm" or "abdominal aorta aneurysm"):ti,ab,kw (Word variations have been searched) |
| CINAHL – 9 May |
| ((Pioglitazone OR thiazolidinedione OR thiazolidinediones OR glitazone OR glitazones OR 2,4-thiazolidinedione OR 2,4-thiazolidinediones OR Actos OR AD4833 OR U72107A OR “Peroxisome proliferator-activated receptor gamma agonist” OR “Peroxisome proliferator-activated receptor gamma” OR “PPAR gamma” OR PPARgamma OR PPAR-gamma OR PPARG OR NR1C3) and (“cardiovascular disease” or "cardiovascular event" or “cardiovascular disorder” or “cardiovascular outcome” or “vascular risk” or “cardiovascular risk” or "coronary disease" or "coronary ischemia" or "coronary ischaemia" or "coronary artery disease" or "coronary heart disease" or "myocardial infarction" or MI or "heart infarction” or stroke or poststroke or post-stroke or "cerebrovascular disease" or "cerebrovascular event" or "cerebrovascular ischemia" or "cerebrovascular ischaemia" or "cerebrovascular accident" or "cerebrovascular hemorrhage" OR “cerebrovascular disorders” OR “cerebrovascular trauma” OR “cerebral hematoma” OR “brain hematoma” OR hematom OR "cerebrovascular infarction” OR “intracranial arterial diseases” OR "cerebral disease" OR "cerebral event" OR "cerebral ischemia" OR "cerebral ischaemia" OR "cerebral accident" OR "cerebral hemorrhage" OR "cerebral haemorrhage" OR “intracranial hemorrhage” OR “intracranial haemorrhage” OR "cerebral infarction" OR "brain infarction" OR "brain hemorrhage" OR "brain haemorrhage" OR "brain ischemia" OR "brain ischaemia" OR “Intracranial vasospasm” OR vasospasm OR "transient ischemic attack" OR "transient ischaemic attack" OR TIA OR TIAs OR “carotid artery diseases”OR “peripheral artery disease" OR "peripheral arterial disease" OR "peripheral artery occlusive disease" OR PAD OR atherosclerosis OR arterial calcification OR atherogenesis OR “percutaneous coronary intervention" OR "percutaneous transluminal angioplasty" OR "percutaneous transluminal coronary angioplasty" OR "coronary artery bypass graft" OR "carotid endarterectomy" OR "carotid artery stenting" OR death OR mortality OR survival OR "ankle brachial index" OR "ankle brachial ratio" OR "arterial stiffness" OR "vascular stiffness"OR "pulse wave analysis" OR "intima media thickness" OR "pulse velocity" OR "pulse wave velocity" OR "augmentation index" OR "aortic calcification" OR Embolism OR thrombosis OR “Abdominal aortic aneurysm” OR “abdominal aorta aneurysm”)) OR ((Pioglitazone OR thiazolidinedione OR thiazolidinediones OR glitazone OR glitazones OR 2,4-thiazolidinedione OR 2,4-thiazolidinediones OR Actos OR AD4833 OR U72107A OR “Peroxisome proliferator-activated receptor gamma agonist” OR “Peroxisome proliferator-activated receptor gamma” OR “PPAR gamma” OR PPARgamma OR PPAR-gamma OR PPARG OR NR1C3) and (“Randomized controlled trial” OR “controlled clinical trial” OR randomized OR placebo OR “clinical trials as topic” OR randomly OR trial OR “random allocation” OR “control groups” OR RCT OR RCTs)) |
| Embase – 10 May |
| 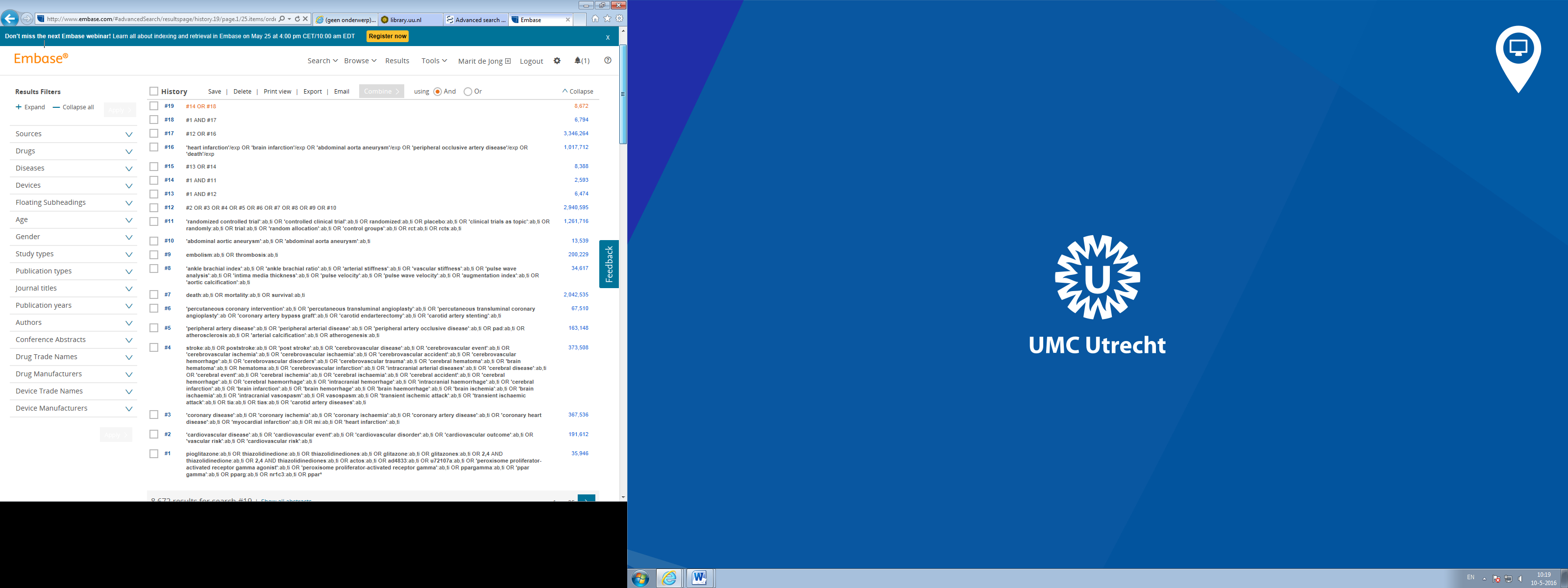 |

Search strategy for Pubmed, Cochrane Library, CINAHL and Embase summarized.

**Additional Table S2:** Details on data extraction

| Data extraction |
| --- |
| From each study, the following data was extracted: surname first author, year of publication, country, study design, study population, sex distribution, mean age, number of patients in intervention and control group, type of intervention, dosage of intervention, type of control, additional treatment in intervention and control group, duration of treatment, duration of follow-up, BMI, hypertension, smoking, HbA1c, LDL, cholesterol, lipid lowering medication, blood pressure lowering medication and predetermined outcomes of interest (MACE (as defined in the study), (non-fatal and fatal) MI, (non-fatal and fatal) stroke, cardiovascular mortality and all-cause mortality) including outcome definitions. |

MACE = major adverse cardiac/cardiovascular events; MI = myocardial infarction.

**Additional Table S3:** Additional study characteristics

| Study,  Year  (Name) | Country | Design | Population | | N  (i/c) | Sex distr. women (%) i/c | Mean age – age distr.  i/c  (Mean ± SD) | Intervention (mg/d) vs. control (mg/d) | Follow-up |
| --- | --- | --- | --- | --- | --- | --- | --- | --- | --- |
| Hong,  2015 [1] | Korea | RCT, multi-center | DM II + CAD + PCI | | 36  36 | 47.2  41.7 | 58.3±11.8  60±12.7 | Pio (15-30) vs. control, not further specified | 9 Months§ |
| Kaneda,  2009 [2] | Japan | Unclear | DM II or non-DM II + STEMI + PCI | | 48  48 | 25  25 | 67±12  67±14 | Pio (15-30) vs. control, not further specified | 6 Months# |
| Kernan,  2016  (IRIS) [3] | US | RCT, multi-center | IR + TIA/stroke | | 1.939  1.937 | 33.3  35.7 | 63.5±10.6  63.5±10.7 | Pio (45) vs. matching placebo tablets | 5 Years  (4.8 years*)# |
| Lee,  2013 [4] | Korea | RCT, single center | DM II + CAD + PCI | | 60  61 | 28.3  24.6 | 60.3±9.5  61.9±8.8 | Pio (15) vs. placebo, not further specified | 12 Months# |
| Nishio,  2006 [5] | Japan | RCT, single center | DM II + CAD + PCI | | 26  28 | 26.9  28.6 | 66.2±8.6  67.5±10.3 | Pio (30) vs. control, not further specified | 6 Months§ |
| Nissen,  2008  (PERI-SCOPE) [6] | US | RCT, multi-center | DM II + CAD | | 274  273 | 31.1  34.1 | 60.0±9.4  59.7±9.1 | Pio (15-45) vs. Glimepiride (1-4) | 18 Months# |
| Suryadevara,  2012[7] | US | Cross-over RCT, single center | DM II + CAD | 15  16 | | 53.3 | 62.5±7.1 | Pio (30) vs. matching placebo tablets | 2 Weeks# |
| Takagi,  2009  (POPPS) [8] | Japan & US | RCT, multi-center | DM II + symptomatic heart disease + PCI | | 48  49 | 17  14 | 64.0±8.8  62.4±9.8 | Pio (30) vs. control, not further specified | 12 Months§ |
| Tanaka,  2015  (J-SPIRIT)  [9] | Japan | RCT, multi-center | AGTT + TIA/ischemic stroke | | 63  57 | 22.2  26.3 | 68.1 (48-82)  68.8 (40-89) | Pio (15-30) vs. not receiving pioglitazone | 5 years  (2.8 years*)# |
| PROactive,  2005 [10] | UK | RCT, multi-center | DM II + macrovascular disease | | 2.605  2.633 | 33  34 | 61.9±7.6  61.6±7.8 | Pio (15-45) vs. matching placebo tablets | 48 Months  (34.5 Months†)# |

Data given as % or mean ± SD. RCT = randomized controlled trial; DM II = diabetes mellitus type II; CAD = coronary artery disease; PCI = percutaneous coronary intervention; STEMI = ST-elevated myocardial infarction; IR = insulin resistance; TIA = transient ischemic attack; AGTT = abnormal glucose tolerance test; i/v = intervention vs. control; Pio = pioglitazone; *median; †mean. #Duration of follow-up and treatment are identical; § duration of follow-up and treatment are most likely identical, but not specifically mentioned by the individual paper.

**Additional Table S4:** Additional study characteristics – modifiable risk factors + medication.

|  | Hong,  2015 [1] | Kaneda,  2009 [2] | Kernan,  2016 [3] | Lee,  2013 [4] | Nishio,  2006 [5] | Nissen,  2008 [6] | Suryadevara,  2012* [7] | Takagi,  2009[8] | Tanaka,  2015 [9] | PROactive [10],[11],[12] |
| --- | --- | --- | --- | --- | --- | --- | --- | --- | --- | --- |
| BMI (kg/m^2^)  (Mean ± SD) | 24.6±3.9  24.4±3.5 | Not reported | 29.9±5.6  30.0±5.3 | 24.0±3.0  23.8±3.2 | 24.6±3.9  24.6±3.5 | 32.1±5.3  32.0±5.2 | 37.8±7.6 | Not reported | 23.8±3.4  24.7±3.1 | 30.7±4.7  31.0±4.8 |
| HbA1c (mmol/L)  (Mean ± SD) | 7.4±1.6  7.5±1.9 | 6.1±1.4  5.9±1.1 | 5.8±0.4  5.8±0.4 | 8.1±1.6  7.4±1.8 | 7.7±2.2  6.9±1.6 | 7.4±1.0  7.4±1.0 | 7.9±1.8 | 7.5±1.8  7.0±1.5 | 6.0±0.4  5.9±0.4 | 7.8(7-8.9)  7.9(7.1-8.9§ |
| Smoking % | 25  19 | 40  29 | Not reported | 50  40 | 23  32 | 12  19 | 20 | 50  53 | 56  56 | 13  14 |
| Hypertension  % | 39  36 | 44  52 | 71  72 | 55  59 | 38  46 | 83  92 | 100 | 67  53 | 79  84 | 75  76† |
| Systolic blood pressure (mmHg) | Not reported | Not reported | 133.2±17.7  133.0±17.3 | Not reported | 126,9±19.4  131.7±16.9 | 127.8±16.6  126.6±17.1 | Not reported | Not reported | 140.7±17.7  137.7±14.7 | 144±18  143±18 |
| LDL  (mmol/L)  (Mean ± SD) | 3.9±1.7  4.1±2 | 3.6±0.8  3±0.7 | 2.3±0.8  2.3±0.8 | 3.2±1  2.7±1.1 | 3.2±0.8  3±0.6 | 2.4± 0.8  2.4± 0.9 | Not reported | 3.1±1  3.2±0.8 | 3.1±1.1  3±0.7 | 2.9 (2.3 -3.5)  2.9 (2.3-3.5)§ |
| Total cholesterol  (mmol/L)  (Mean ± SD) | 5.5±1.6  5.7±1.2 | 5.5±1  5.3±0.8 | Not reported | 5.3±1.1  4.7±1.5 | 5.1±1.2  4.8±0.9 | 4.2+0.9  4.2+1 | Not reported | 5.1±1.2  5.2±1 | Not reported | Not reported |
| *Lipid lowering medication* |  |  |  |  |  |  |  |  |  |  |
| Statin % | Not reported | 71  58 | 83  82 | 73  74 | 62  46 | 82  82 | 100 | 65  53 | 46  42 | 43  43 |
| Other lipid lowering agents % | Not reported | Not reported | Not reported | Not reported | Not reported | 5  6 | Not reported | Not reported | Not reported | 10  11 |
| *Blood pressure lowering agents* |  |  |  |  |  |  |  |  |  |  |
| ACEI % | 8  11 | Not reported | Not reported | Not reported | 20  18 | Not reported | Not reported | 38  22 | 0  0 | 63  63 |
| ARB % | 30  25 | Not reported | Not reported | Not reported | 54  39 | Not reported | Not reported | 27  31 | 54  63 | 7  7 |
| ACEI or ARB % | Not reported | 75  71 | 56  55 | Not reported | Not reported | 80  84 | 87 | Not reported | Not reported | Not reported |
| CCB % | 33  39 | Not reported | Not reported | Not reported | Not reported | Not reported | 40 | 25  22 | 41  49 | 34  37 |
| B-blocker % | 25  17 | 50  46 | 32  32 | Not reported | 20  14 | 76  77 | Not reported | 38  39 | 3  11 | 55  54 |
| Diuretics % | 14  8 | 25  15 | 30  28 | Not reported | Not reported | Not reported | Not reported | 10  20 | 5  5 | Not reported |
| Thiazide diuretics % | Not reported | Not reported | Not reported | Not reported | Not reported | Not reported | Not reported | Not reported | Not reported | 15  16 |
| Loop diuretics % | Not reported | Not reported | Not reported | Not reported | Not reported | Not reported | Not reported | Not reported | Not reported | 14  14 |
| Spironolactone  % | Not reported | 25  27 | Not reported | Not reported | Not reported | Not reported | Not reported | Not reported | Not reported | Not reported |
| Nitrates % | 36  47 | Not reported | Not reported | Not reported | Not reported | Not reported | 40 | 33  35 | Not reported | 39  40 |
| Nicorandil % | 8  8 | Not reported | Not reported | Not reported | Not reported | Not reported | Not reported | 35  31 | Not reported | Not reported |

Data given as % or mean ± SD. ACEI = angiontensin-converting enzyme inhibitor; ARB = angiotensin II receptor blocker; CCB = calcium channel blocker; *baseline characteristics of participants that finished both study groups; †history of hypertension; § median IQR.

**Additional Table S5:** Risk of bias summary.

|  | Random sequence generation? (selection bias) | Allocation concealment?  (selection bias) | Blinding of participants and personnel? (performance bias) | Blinding of outcome assessment? (detection bias) | Incomplete outcome data? (attrition bias) | Selective reporting? (reporting bias) | Other bias |
| --- | --- | --- | --- | --- | --- | --- | --- |
| Hong, 2015[1] | Unclear (insufficient information) | Unclear (insufficient information) | High risk (single-blinded) | Low risk (outcome assessors were blinded despite single-blinded design) | Low risk | Low risk | Low risk |
| Kaneda, 2009[2] | Unclear (insufficient information) | Unclear (insufficient information) | High risk (single-blinded) | High risk (single-blinded, insufficient information) | Low risk | Low risk | Low risk |
| Kernan, 2016[3] | Low risk | Low risk | Low risk | Low risk | Low risk | Low risk | Low risk |
| Lee, 2013[4] | Unclear (insufficient information) | Unclear (insufficient information) | Unclear (insufficient information) | Unclear (insufficient information, CAG/IVUS data was reviewed blinded, clinical data not reported) | Low risk | Low risk | Low risk |
| Nishio, 2006[5] | Unclear (insufficient information) | Unclear (insufficient information) | High risk (single-blinded) | High risk (single-blinded, insufficient information) | Low risk | Low risk | Low risk |
| Nissen, 2008[6] | Low risk | Low risk | Low risk | Low risk | Low risk | Low risk | Low risk |
| Suryadevara, 2012[7] | Low risk | Low risk | Low risk | Low risk | High risk (high loss to follow-up, no intention-to-treat analysis) | Low risk | Low risk |
| Takagi, 2009[8] | Unclear (insufficient information) | Unclear (insufficient information) | High risk (open-label design) | High risk (open-label design, insufficient information) | Low risk | Low risk | Low risk |
| Tanaka 2015 | Unclear (insufficient information) | Unclear (insufficient information) | High risk (open-label design) | High risk (open-label design, insufficient information) | Low risk | Low risk | Low risk |
| PROactive, 2005[10],  [11],[12] | Low risk | Low risk | Low risk | Low risk | Low risk | Low risk | Low risk |

This table summarizes the risk of bias per individual study included in the meta-analysis.

**Additional Table S6:** Absolute risk reduction, numbers needed to treat/harm and relative risk

| Outcome of interest | Absolute Risk Reduction | Number Needed to Treat | RR |
| --- | --- | --- | --- |
| MACE 1 | 601/5063 – 463/5032 = 0.027 | 1/0.026 = 38.6 | 0.74 |
| MACE 2 | 320/2942 – 262/2911 = 0.019 | 1/0.019 = 53.3 | 0.83 |
| Myocardial infarction | 233/5065 – 177/5032 = 0.011 | 1/0.011 = 92.4 | 0.77 |
| Stroke | 269/4900 – 217/4877 = 0.010 | 1/0.010 = 96.1 | 0.81 |
| Stroke recurrence | 212/2492 – 157/2488 = 0.022 | 1/0.022 = 45.5 | 0.69 |
| Outcome of interest | Absolute Risk Increase | Number needed to Harm | RR |
| Heart failure | 277/5031 – 364/5009 = 0.018 | 1/0.018 = 56.7 | 1.33 |

MACE 1 = major adverse cardiac/cardiovascular disease as defined in the included studies; MACE 2 = composite of nonfatal stroke, nonfatal myocardial infarction + cardiovascular mortality; RR = relative risk.

**Additional Table S7:** Sensitivity analyses

|  | M-H  (95% CI) |  | M-H  (95% CI) |  | M-H  (95% CI) |  | M-H  (95% CI) |  | M-H  (95% CI) |
| --- | --- | --- | --- | --- | --- | --- | --- | --- | --- |
| Major adverse cardiovascular events:  RR, RE | 0.74  (0.60-0.92) | Myoycardial infraction:  RR, RE | 0.66  (0.48-0.91) | Stroke:  RR, RE | 0.81  (0.68-0.96) | Death:  RR, RE | 0.94  (0.81-1.09) | Heart failure:  RR, RE | 1.33  (1.14-1.54) |
| Major adverse cardiovascular events:  RR, FE | 0.78  (0.69-0.87) | Myoycardial infraction:  RR, FE | 0.66  (0.48-0.91) | Stroke:  RR, FE | 0.81  (0.68-0.96) | Death:  RR, FE | 0.93  (0.81-1.08) | Heart failure:  RR, FE | 1.32  (1.14-1.54) |
| Major adverse cardiovascular events:  OR, RE | 0.68  (0.50-0.91) | Myoycardial infraction:  OR, RE | 0.65  (0.46-0.91) | Stroke:  OR, RE | 0.80  (0.66-0.96) | Death:  OR, RE | 0.93  (0.80-1.09) | Heart failure:  OR, RE | 1.35  (1.15-1.59) |
| Major adverse cardiovascular events:  OR, FE | 0.75  (0.66-0.85) | Myoycardial infraction:  OR, FE | 0.65  (0.46-0.90) | Stroke:  OR, FE | 0.80  (0.66-0.96) | Death:  OR, FE | 0.93  (0.79-1.09) | Heart failure:  OR, FE | 1.35  (1.15-1.59) |

RE = random effects model; FE = fixed effects model; RR = relative risk; OR = odds ratio; M-H = Mantel-Haenszel method; 95% CI = 95% confidence interval.

**Additional Table S8:** Subgroup analyses

|  | Non-DM II + DM II (RR, RE – 95% CI) | DM II only (RR, RE – 95% CI) | Studies excluded  (No DM II patients) | Studies Included  (DM II patients) |
| --- | --- | --- | --- | --- |
| Major adverse cardiovascular events | 0.74 (0.60-0.92) I^2^ = 35% | 0.58 (0.35-0.98)  I^2^ = 53% | Kaneda 2009[2],  Kernan 2016[3] | Hong 2015[1], Lee 2013[4], Nishio 2006[5], Nissen 2008[6], Takagi 2009[8], Wilcox 2008[12] |
| Myocardial infarction | 0.77 (0.64-0.93)  I^2^ = 0% | 0.83 (0.66-1.04)  I^2^ = 0% | Kaneda 2009[2], Kernan 2016[3] | Hong 2015[1], Lee 2013[4], Nishio 2006[5], Nissen 2008[6], Takagi 2009[8] |
| Stroke | 0.80 (0.66-0.96)  I^2^ = 0% | 0.81 (0.61-1.07)  I^2^ = 0% | Kernan 2016[3], Tanaka 2015[9] | Nissen 2008[6], Wilcox 2008[12] |
| All-cause mortality | 0.94 (0.81-1.08)  I^2^ = 0% | 0.95 (0.78-1.16)  I^2^ = 0% | Kaneda 2009[2], Kernan 2016[3], Tanaka 2015[9] | Lee 2013[4], Nissen 2008[6], Suryadevara 2012[7], Takagi 2009[8], Wilcox 2008[12] |
| Heart failure | 1.33 (1.09-1.64)  I^2^ = 0% | 1.42 (1.19-1.68)  I^2^ = 0% | Kaneda 2009[2], Kernan 2016[3],  Tanaka 2015[9] | Dormandy 2005[10], Hong 2015[1], Nissen 2008[6], Takagi 2009[8] |

Excluding studies with nondiabetic patients. DM II = diabetes mellitus type II; RR = random effects model; RR = relative risk; 95% CI = 95% confidence interval; I^2^ = % statistical heterogeneity.

**Additional Table S9:** Definitions of major adverse cardiac/cardiovascular events

| Study/ Event | Hong  2015  [1] | Kaneda  2009  [2] | Kernan  2016  [3] | Lee  2013  [4] | Nishio  2006  [5] | Nissen  2008  [6] | Takagi  2009  [8] | Wilcox  2008  [12] |
| --- | --- | --- | --- | --- | --- | --- | --- | --- |
| Death | X | X |  | X | X |  | X |  |
| Cardiac/  cardiovascular mortality |  |  |  |  |  | X |  | X |
| MI | X |  | X | X |  |  | X |  |
| Nonfatal MI |  |  |  |  |  | X |  | X |
| Q-wave MI |  |  |  |  | X |  |  |  |
| Non-Q-wave MI |  |  |  |  | X |  |  |  |
| HF requiring hospitalization |  | X |  |  |  |  |  |  |
| Stroke | X |  | X |  |  |  |  |  |
| Nonfatal stroke |  |  |  |  |  | X |  | X |
| Reinfarction/  Stent thrombosis |  | X |  | X |  |  |  |  |
| Reintervention/  Revasculariza-tion/ TVR/TLR |  |  |  | X | X |  | X |  |
| CABG |  |  |  |  | X |  |  |  |

The individual major adverse cardiac/cardiovascular (MACE 1) composites of the included studies. Hong, 2015: MACE was not reported, but manually calculated. MI = myocardial infarction; HF = heart failure; TVR = target vessel revascularization; TLR = target lesion revascularization; CABG = coronary artery bypass graft.

**Additional Table S10:** Definitions of myocardial infarction

| Study | Definition of myocardial infarction used in the included study |
| --- | --- |
| Hong, 2015[1] | Not reported |
| Kaneda, 2009[2] | ‘Recurrent symptoms with new ST-segment elevation and elevation of cardiac markers to at least twice the upper limit of normal.’ |
| Kernan, 2016[3],[13] | ‘Fatal and non-fatal acute myocardial infarction will be diagnosed according to criteria modified from the 2000 Consensus Conference of the European and American Colleges of Cardiology. Acute myocardial infarction is diagnosed based on symptoms of myocardial ischemia, electrocardiogram changes, and contemporary biochemical markers of myocardial necrosis. An elevated serum concentration of cardiac troponin is a sensitive and specific marker of myocardial necrosis.’ |
| Lee, 2013[4] | ‘Non-ST segment elevation myocardial infarction (NSTE­MI) and ST segment elevation myocardial infarction (STEMI).’ |
| Nishio, 2006[5] | ‘Q wave or non–Q wave myocardial infarction’ |
| Nissen, 2008[6] | Not reported |
| Takagi, 2009[8] | Not reported |
| PROactive[10],[12] | ‘Non-fatal myocardial infarction if the patient survived more than 24 h from onset of symptoms and, in the absence of percutaneous coronary intervention or coronary artery bypass graft, had at least two of: symptoms suggestive of myocardial infarction (ischaemic chest pain or discomfort) lasting 30 min or longer, electrocardiographic evidence of myocardial infarction, or raised cardiac serum markers; or after percutaneous coronary intervention or coronary artery bypass graft the patient had electrocardiographic evidence of myocardial infarction.’ Silent myocardial infarction was defined as new Q waves on two contiguous leads or R-wave reduction in the precordial leads without a change in axis deviation.’ |

This table displays the definition of myocardial infarction, used by the individual studies included in the meta-analysis.

**Additional Table S11:** Definitions of Stroke

| Study | Definition of stroke used in the included study |
| --- | --- |
| PROactive[12] | ‘An acute focal neurologic deficit lasting for >24 hours or resulting in death within 24 hours of the onset of symptoms, which was diagnosed as being due to a cerebral lesion of vascular origin, but excluding subarachnoid hemorrhage.’ |
| Tanaka, 2015[9] | ‘Cerebrovascular events were considered to be definite lesions related to neurological symptoms and signs, which correlated with a new lesion on CT or MRI.’ |
| Kernan, 2016[3],[13] | ‘Acute neurological event with focal signs or symptoms lasting more than 24 hours which represent a focal loss of brain function that can be attributed to a disturbance in one vascular distribution and for which no other cause is found. In addition, there must be at least a one-point increase in the NIH stroke scale in a previously normal section, or an appropriate new or extended abnormality seen on CT or MRI. IRIS will count non-traumatic intracerebral and subarachnoid hemorrhage as outcomes in addition to ischemic events. Subdural and epidural hematoma are not included as part of the primary stroke outcome.’ |
| Hong, 2015[1] | Not reported |

This table displays the definition of stroke, used by the individual studies included in the meta-analysis.

**Additional Table S12:** Definitions of heart failure

| Study | Definition of heart failure used in the included study |
| --- | --- |
| Kernan, 2016[3] | ‘Heart failure needing hospitalization or causing death’ |
| PROactive[10] | ‘Any report of heart failure’ |
| Nissen, 2008[6] | ‘Heart failure needing hospitalization’ |
| Takagi, 2009[8] | ‘Heart failure needing hospitalization’ |
| Tanaka, 2015[9] | ‘Complication of heart failure causing drug discontinuation’ |
| Hong, 2015[1] | ‘New onset heart failure’ |
| Kaneda, 2009[2] | ‘Any heart failure’ |

This table displays the definition of heart failure, used by the individual studies included in the meta-analysis.

**Figure S1.** Funnel plots

**
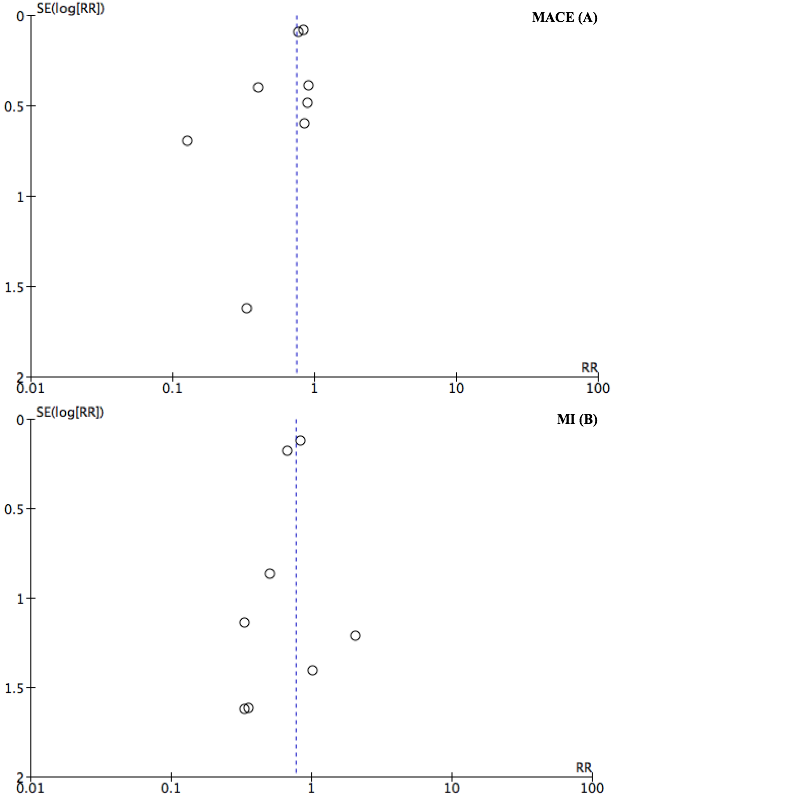
**


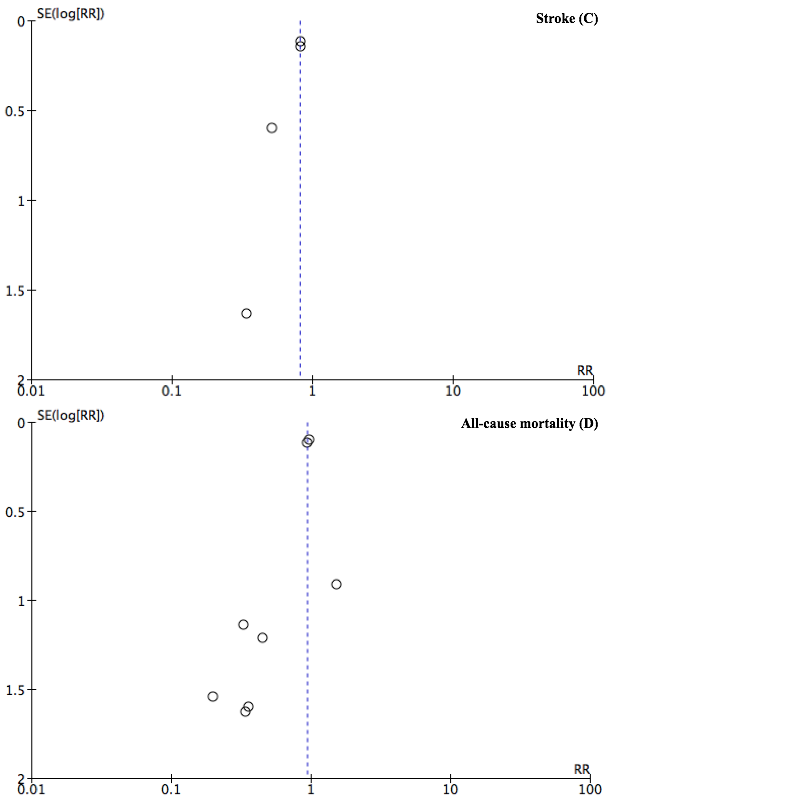


Funnel plots for major adverse cardiac/cardiovascular events (A), myocardial infarction (B), stroke (C), and all-cause mortality (D). MACE=major adverse cardiac/cardiovascular events; MI=myocardial infarction.

**Figure S2.** Analyses on major adverse cardiac/cardiovascular events


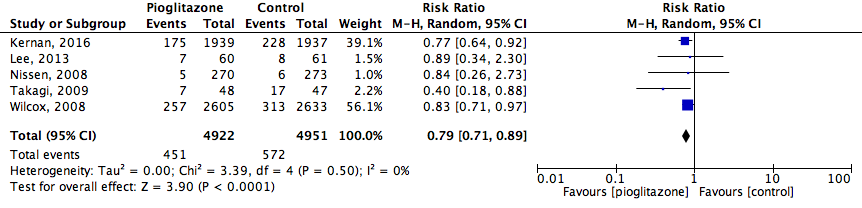


Only studies with a follow-up duration of 12 months or longer were included.

**Figure S3.** Analyses on major adverse cardiac/cardiovascular events 2


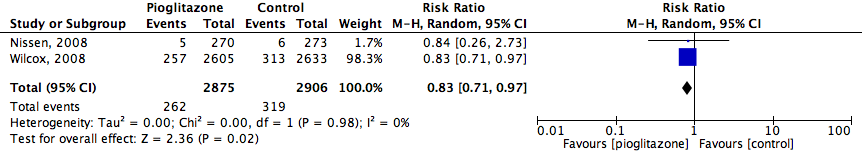


Only studies with a follow-up duration of 12 months or longer were included.

**Figure S4.** Analyses on myocardial infarction.


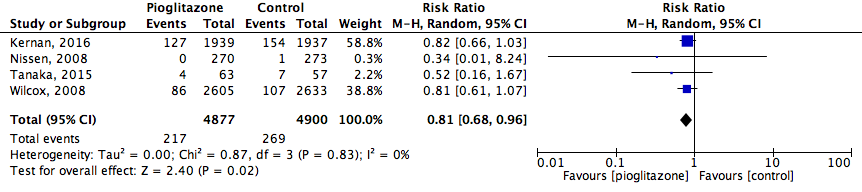


Only studies with a follow-up duration of 12 months or longer were included.

**Figure S5.** Analyses on all-cause mortality


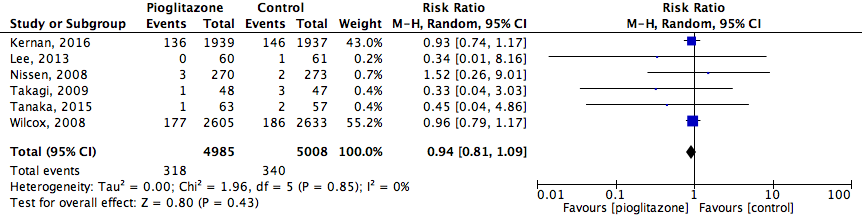


Only studies with a follow-up duration of 12 months or longer were included.

**Figure S6.** Analyses on heart failure


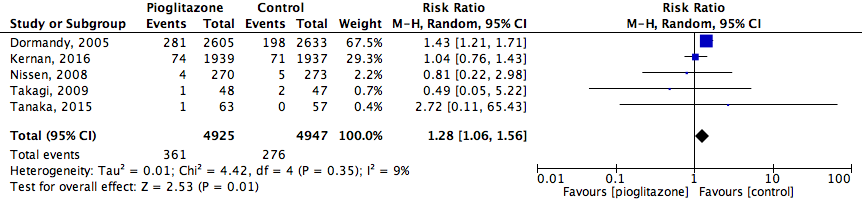


Only studies with a follow-up duration of 12 months or longer were included.

**References supplemental data**

[1] S.J. Hong, S.C. Choi, J.Y. Cho, et al. Pioglitazone increases circulating microRNA-24 with decrease in coronary neointimal hyperplasia in type 2 diabetic patients-optical coherence tomography analysis, Circ. J. 79 (2015) 880–8. doi:10.1253/circj.CJ-14-0964.

[2] H. Kaneda, T. Shiono, Y. Miyashita, et al. Efficacy and safety of pioglitazone in patients with ST elevation myocardial infarction treated with primary stent implantation, Heart. 95 (2009) 1079–84. doi:10.1136/hrt.2008.162842.

[3] W.N. Kernan, C.M. Viscoli, K.L. Furie, et al. Pioglitazone after Ischemic Stroke or Transient Ischemic Attack, N. Engl. J. Med. 374 (2016) 1321–31. doi:10.1056/NEJMoa1506930.

[4] H.W. Lee, H.C. Lee, B.W. Kim, et al. Effects of low dose pioglitazone on restenosis and coronary atherosclerosis in diabetic patients undergoing drug eluting stent implantation, Yonsei Med. J. 54 (2013) 1313–20. doi:10.3349/ymj.2013.54.6.1313.

[5] K. Nishio, M. Sakurai, T. Kusuyama, et al. A randomized comparison of pioglitazone to inhibit restenosis after coronary stenting in patients with type 2 diabetes, Diabetes Care. 29 (2006) 101–6. doi:10.2337/diacare.29.01.06.dc05-1170.

[6] S.E. Nissen, S.J. Nicholls, K. Wolski, et al. Comparison of pioglitazone vs glimepiride on progression of coronary atherosclerosis in patients with type 2 diabetes: the PERISCOPE randomized controlled trial, JAMA. 299 (2008) 1561–73. doi:10.1001/jama.299.13.1561.

[7] S. Suryadevara, M. Ueno, A. Tello-Montoliu, et al. Effects of pioglitazone on platelet P2Y12-mediated signalling in clopidogrel-treated patients with type 2 diabetes mellitus, Thromb. Haemost. 108 (2012) 930–6. doi:10.1160/TH12-06-0397.

[8] T. Takagi, H. Okura, Y. Kobayashi, et al. A Prospective, Multicenter, Randomized Trial to Assess Efficacy of Pioglitazone on In-Stent Neointimal Suppression in Type 2 Diabetes: POPPS (Prevention of In-Stent Neointimal Proliferation by Pioglitazone Study), JACC Cardiovasc. Interv. 2 (2009) 524–531. doi:10.1016/j.jcin.2009.04.007.

[9] R. Tanaka, K. Yamashiro, Y. Okuma, et al. Effects of Pioglitazone for Secondary Stroke Prevention in Patients with Impaired Glucose Tolerance and Newly Diagnosed Diabetes: The J-SPIRIT Study, J. Atheroscler. Thromb. 22 (2015) 1305–16. doi:10.5551/jat.30007.

[10] J.A. Dormandy, B. Charbonnel, D.J. Eckland, et al. Secondary prevention of macrovascular events in patients with type 2 diabetes in the PROactive Study (PROspective pioglitAzone Clinical Trial In macroVascular Events): a randomised controlled trial, Lancet. 366 (2005) 1279–1289. doi:10.1016/S0140-6736(05)67528-9.

[11] R. Wilcox, M.-G. Bousser, D.J. Betteridge, et al. Effects of Pioglitazone in Patients With Type 2 Diabetes With or Without Previous Stroke: Results From PROactive (PROspective pioglitAzone Clinical Trial In macroVascular Events 04), Stroke. 38 (2007) 865–873. doi:10.1161/01.STR.0000257974.06317.49.

[12] R. Wilcox, S. Kupfer, E. Erdmann, Effects of pioglitazone on major adverse cardiovascular events in high-risk patients with type 2 diabetes: Results from PROspective pioglitAzone Clinical Trial In macro Vascular Events (PROactive 10), Am. Heart J. 155 (2008) 712–717. doi:10.1016/j.ahj.2007.11.029.

[13] L.M. Brass, K.L. Furie, M. Gorman, et al. Stand-Alone Protocol. The insulin resistance intervention after stroke trial (IRIS). A randomized, placebo-controlled trial of pioglitazone compared with placebo, for prevention of stroke and myocardial infarction after ischemic stroke and transient ischemic attack. http://citeseerx.ist.psu.edu/viewdoc/download?doi=10.1.1.465.2402&rep=rep1&type=pdf, 2011 (accessed 10.05.16).
